# (*E*)-(2,4-Dichloro­phen­yl)[2-hydr­oxy-6-(methoxy­imino)cyclo­hex-1-en­yl]methanone

**DOI:** 10.1107/S1600536809001883

**Published:** 2009-01-23

**Authors:** Guang-Dong Huang, Jian-Wei Zou, Wen-Na Zhao, Shu-Min Zhao

**Affiliations:** a Department of Chemistry, Zhejiang University, Hangzhou 310027, People’s Republic of China; b Ningbo Institute of Technology, Zhejiang University, Ningbo, Zhejiang 315100, People’s Republic of China

## Abstract

The title compound, C_14_H_13_Cl_2_NO_3_, was obtained as the product of an attempted synthesis of herbicidally active compounds containing oxime ether and cyclo­hexenone groups. In the crystal structure, the mol­ecule adopts an endocyclic enol tautomeric form and the cyclo­hexene ring adopts a distorted envelope form. The oxime ether group has an *E* configuration, with the meth­oxy group *anti* to the *ortho*-chloro substitutent. Intra­molecular O—H⋯O and inter­molecular C—H⋯O hydrogen bonds are found in the crystal structure.

## Related literature

For the structure of 5-chloro-2-methyl­thio-3*H*-indole-3-one 3-oxime *O*-methyl ether, see: Beddoes *et al.* (1992 ▶). For theoretical studies on the tautomerism of benzoyl­cyclo­hexane-1,3-dione and its derivatives, see: Huang *et al.* (2002 ▶). For the potential herbicidal property of the title compound and related compounds, see: Knudsen (1988 ▶). For the chemistry of 2-acyl­cyclo­alkane-1,3-diones, see: Rubinov *et al.* (1999 ▶).
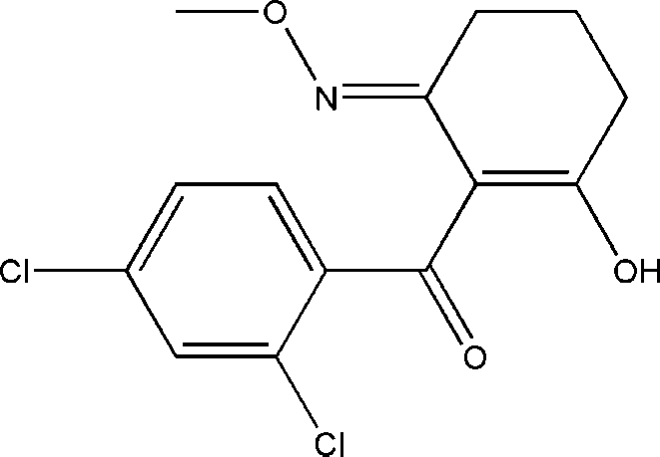


## Experimental

### 

#### Crystal data

C_14_H_13_Cl_2_NO_3_
*M* *_r_* = 314.15
Triclinic, 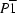

*a* = 8.4096 (17) Å
*b* = 8.9944 (18) Å
*c* = 11.740 (2) Å
α = 68.38 (3)°
β = 74.50 (3)°
γ = 62.32 (3)°
*V* = 726.0 (3) Å^3^
*Z* = 2
Mo *K*α radiation
μ = 0.45 mm^−1^
*T* = 298 (2) K
0.68 × 0.34 × 0.23 mm

#### Data collection

Bruker APEXII CCD diffractometer
Absorption correction: multi-scan (*SADABS*; Sheldrick, 2001 ▶) *T*_min_ = 0.748, *T*_max_ = 0.903
6630 measured reflections
3247 independent reflections
2162 reflections with *I* > 2σ(*I*)
*R*_int_ = 0.021

#### Refinement

*R*[*F* ^2^ > 2σ(*F* ^2^)] = 0.049
*wR*(*F* ^2^) = 0.144
*S* = 1.08
3247 reflections
233 parameters
H atoms treated by a mixture of independent and constrained refinement
Δρ_max_ = 0.35 e Å^−3^
Δρ_min_ = −0.40 e Å^−3^

### 

Data collection: *SMART* (Bruker, 2003 ▶); cell refinement: *SAINT* (Bruker, 2003 ▶); data reduction: *SAINT*; program(s) used to solve structure: *SHELXS97* (Sheldrick, 2008 ▶); program(s) used to refine structure: *SHELXL97* (Sheldrick, 2008 ▶); molecular graphics: *SHELXTL* (Sheldrick, 2008 ▶); software used to prepare material for publication: *SHELXL97*.

## Supplementary Material

Crystal structure: contains datablocks global, I. DOI: 10.1107/S1600536809001883/si2144sup1.cif

Structure factors: contains datablocks I. DOI: 10.1107/S1600536809001883/si2144Isup2.hkl

Additional supplementary materials:  crystallographic information; 3D view; checkCIF report

## Figures and Tables

**Table 1:** Hydrogen-bond geometry (Å, °)

*D*—H⋯*A*	*D*—H	H⋯*A*	*D*⋯*A*	*D*—H⋯*A*
O2—H10⋯O1	0.91 (3)	1.64 (3)	2.485 (2)	152 (3)
C4—H1⋯O1^i^	0.99 (2)	2.51 (2)	3.347 (3)	143 (2)
C2—H3⋯O2^ii^	0.91 (4)	2.57 (3)	3.438 (3)	160 (3)

Symmetry codes: (i) 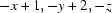; (ii) 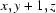.

**Fig. 1.**
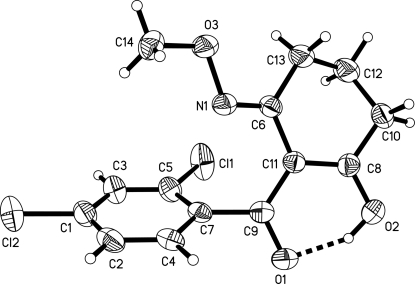
Perspective view of the title complex with atom numbering scheme. Thermal ellipsoids are shown at 30% probability level.

**Fig. 2.**
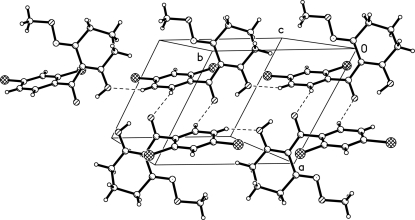
One-dimensional supramolecular structure showing intermolecular C—H···O hydrogen bonding contacts.

## supplementary crystallographic information

### Comment

2-Acylcycloalkanone derivatives, either natural or synthetic products, have been widely studied because of their versatile biological activity (Rubinov *et al.* 1999). The title compound, which was reported previously as a potential herbicide (Knudsen, 1988), was synthesized using 3-chloro-2-(2,4-dichlorobenzoyl)cyclohex-2-enone and methoxyamine hydrochloride as reactants and triethylamine as a catalyst. Herin we report its crystal structure with an attempt to understand better the structure-activity relationship of this class of compounds.

The title compound consists of 2,4-dichlorobenzoyl and cyclohexenone oxime *O*-methyl ether moieties. It has several possible tautomers in solution due to the existence of a 1,3-dione structure (Huang *et al.* 2002). The title molecule adopts an endocyclic enol tautomeric form in the crystal structure, *i.e.* the carbonyl group of the cyclohexenone unit is enolized. The cyclohexene ring adopts a distorted envelope form. The oxime ether is in an *E* configuration, with the methoxy group being *anti* to the substitutent group at the C-11 position (Fig. 1). The bond length of C6=N1 is 1.284 (3) Å and the C=N—O angle is 110.89 (17)°, which is close to the value in 5-chloro-2-methylthio-3*H*-indole-3-one 3-oxime *O*-methyl ether, (111.3 (5)°) (Beddoes *et al.*, 1992), showing that the C6=N1 bond is conjugated with the C8=C11 and C9=O1 bonds.

There is a strong intramolecular hydrogen bond, O2—H10···O1 (Table 1) and, as a result, a *pseudo*-six-membered ring (C8—O2—H10···O1—C9—C11) is formed in the structure (Fig. 1). The torsional angle of O1—C9—C8—C11 is 11.9 (3)°. In addition, two weak intermolecular C—H···O hydrogen bonding contacts, which form columns along the *b* axis, are found in the packing structure (Table 1 and Fig. 2).

### Experimental

A mixture of 2-(2,4-dichlorobenzoyl)cyclohexen-1,3-dione (0.57 g, 2 mmol), methoxyamine hydrochloride (0.18 g, 2.2 mmol) and anhydrous sodium acetate (0.2 g, 2.4 mmol) was stirred in methanol (30 ml) at room temperature for 16 h. The mixture was diluted with 100 ml of water and extracted with 30 ml e thyl acetate three times. The combined organic layer was dried with anhydrous magnesium sulfate and purified by column chromatography (ethyl acetate:petroleum ether = 1:12) to afford the title compound (70% yield). A crystal suitable for X-ray analysis was obtained by recrystallization of the product with acetone/pentane (1:10) at room temperature over a period of 3 d.

### Refinement

All H atoms were located in difference Fourier maps and refined independently with isotropic displacement parameters.

### Crystal data

**Table:**

C_14_H_13_Cl_2_NO_3_	*Z* = 2
*M**_r_* = 314.15	*F*(000) = 324
Triclinic, *P*1	*D*_x_ = 1.437 Mg m^−^^3^
Hall symbol: -P 1	Mo *K*α radiation, λ = 0.71073 Å
*a* = 8.4096 (17) Å	Cell parameters from 3247 reflections
*b* = 8.9944 (18) Å	θ = 3.2–27.5°
*c* = 11.740 (2) Å	µ = 0.45 mm^−^^1^
α = 68.38 (3)°	*T* = 298 K
β = 74.50 (3)°	Block, green
γ = 62.32 (3)°	0.68 × 0.34 × 0.23 mm
*V* = 726.0 (3) Å^3^	

### Data collection

**Table:**

Bruker APEXII CCD diffractometer	3247 independent reflections
Radiation source: fine-focus sealed tube	2162 reflections with *I* > 2σ(*I*)
graphite	*R*_int_ = 0.021
Detector resolution: 8.40 pixels mm^-1^	θ_max_ = 27.5°, θ_min_ = 3.2°
ω scans	*h* = −10→9
Absorption correction: multi-scan (*SADABS*; Sheldrick, 2001)	*k* = −11→10
*T*_min_ = 0.748, *T*_max_ = 0.903	*l* = −15→15
6630 measured reflections	

### Refinement

**Table:**

Refinement on *F*^2^	Primary atom site location: structure-invariant direct methods
Least-squares matrix: full	Secondary atom site location: difference Fourier map
*R*[*F*^2^ > 2σ(*F*^2^)] = 0.049	Hydrogen site location: inferred from neighbouring sites
*wR*(*F*^2^) = 0.144	H atoms treated by a mixture of independent and constrained refinement
*S* = 1.08	*w* = 1/[σ^2^(*F*_o_^2^) + (0.0701*P*)^2^ + 0.0941*P*] where *P* = (*F*_o_^2^ + 2*F*_c_^2^)/3
3247 reflections	(Δ/σ)_max_ < 0.001
233 parameters	Δρ_max_ = 0.35 e Å^−^^3^
0 restraints	Δρ_min_ = −0.40 e Å^−^^3^

### Special details

**Table:**

Geometry. All e.s.d.'s (except the e.s.d. in the dihedral angle between two l.s. planes) are estimated using the full covariance matrix. The cell e.s.d.'s are taken into account individually in the estimation of e.s.d.'s in distances, angles and torsion angles; correlations between e.s.d.'s in cell parameters are only used when they are defined by crystal symmetry. An approximate (isotropic) treatment of cell e.s.d.'s is used for estimating e.s.d.'s involving l.s. planes.
Refinement. Refinement of *F*^2^ against ALL reflections. The weighted *R*-factor *wR* and goodness of fit *S* are based on *F*^2^, conventional *R*-factors *R* are based on *F*, with *F* set to zero for negative *F*^2^. The threshold expression of *F*^2^ > σ(*F*^2^) is used only for calculating *R*-factors(gt) *etc*. and is not relevant to the choice of reflections for refinement. *R*-factors based on *F*^2^ are statistically about twice as large as those based on *F*, and *R*- factors based on ALL data will be even larger.

### Fractional atomic coordinates and isotropic or equivalent isotropic displacement parameters (Å^2^)

**Table:**

	*x*	*y*	*z*	*U*_iso_*/*U*_eq_	
Cl1	0.20742 (14)	0.71882 (10)	0.43507 (6)	0.0984 (3)	
Cl2	0.28869 (13)	1.27753 (12)	0.43224 (9)	0.1098 (3)	
O1	0.4465 (2)	0.7560 (2)	0.09971 (15)	0.0676 (4)	
O2	0.3124 (2)	0.5952 (2)	0.05046 (15)	0.0658 (4)	
H10	0.389 (4)	0.641 (4)	0.050 (3)	0.099 (10)*	
O3	−0.25125 (19)	1.1694 (2)	0.24393 (17)	0.0686 (5)	
N1	−0.0704 (2)	1.0779 (2)	0.19908 (16)	0.0551 (4)	
C1	0.2934 (3)	1.1452 (3)	0.3538 (3)	0.0689 (6)	
C2	0.3365 (3)	1.1861 (3)	0.2287 (3)	0.0677 (7)	
H3	0.361 (4)	1.281 (4)	0.184 (3)	0.087 (9)*	
C3	0.2551 (4)	1.0015 (3)	0.4191 (3)	0.0702 (7)	
H6	0.231 (4)	0.968 (4)	0.507 (3)	0.089 (9)*	
C4	0.3381 (3)	1.0831 (3)	0.1661 (2)	0.0583 (5)	
H1	0.370 (3)	1.110 (3)	0.076 (2)	0.064 (7)*	
C5	0.2563 (3)	0.9004 (3)	0.3541 (2)	0.0604 (5)	
C6	−0.0470 (3)	0.9302 (3)	0.19137 (19)	0.0511 (5)	
C7	0.2943 (2)	0.9405 (3)	0.22713 (19)	0.0510 (5)	
C8	0.1559 (3)	0.6921 (3)	0.09904 (19)	0.0539 (5)	
C9	0.2954 (3)	0.8347 (3)	0.15450 (19)	0.0518 (5)	
C10	0.0020 (3)	0.6524 (4)	0.1021 (3)	0.0688 (7)	
H8	−0.056 (4)	0.718 (4)	0.021 (3)	0.113 (11)*	
H11	0.041 (4)	0.528 (4)	0.105 (3)	0.108 (10)*	
C11	0.1366 (3)	0.8193 (3)	0.14735 (18)	0.0485 (5)	
C12	−0.1359 (4)	0.6906 (4)	0.2117 (3)	0.0826 (8)	
H7	−0.244 (4)	0.675 (4)	0.210 (3)	0.100 (9)*	
H14	−0.080 (5)	0.606 (5)	0.301 (4)	0.139 (13)*	
C13	−0.1984 (3)	0.8748 (4)	0.2142 (3)	0.0746 (7)	
H2	−0.259 (4)	0.888 (4)	0.289 (3)	0.086 (9)*	
H15	−0.275 (5)	0.953 (5)	0.131 (4)	0.143 (14)*	
C14	−0.2681 (4)	1.3347 (3)	0.2447 (3)	0.0700 (7)	
H4	−0.178 (4)	1.311 (4)	0.295 (3)	0.101 (10)*	
H5	−0.386 (4)	1.389 (4)	0.281 (3)	0.085 (8)*	
H9	−0.252 (4)	1.405 (4)	0.157 (3)	0.096 (9)*	

### Atomic displacement parameters (Å^2^)

**Table:**

	*U*^11^	*U*^22^	*U*^33^	*U*^12^	*U*^13^	*U*^23^
Cl1	0.1770 (9)	0.0882 (5)	0.0587 (4)	−0.0887 (6)	−0.0179 (4)	−0.0028 (3)
Cl2	0.1302 (7)	0.1061 (6)	0.1376 (8)	−0.0655 (6)	−0.0103 (6)	−0.0629 (6)
O1	0.0478 (9)	0.0763 (11)	0.0799 (11)	−0.0253 (8)	0.0041 (8)	−0.0315 (9)
O2	0.0621 (10)	0.0652 (10)	0.0717 (10)	−0.0211 (9)	−0.0022 (8)	−0.0318 (9)
O3	0.0475 (8)	0.0587 (9)	0.1003 (12)	−0.0155 (7)	−0.0036 (8)	−0.0352 (9)
N1	0.0437 (9)	0.0536 (10)	0.0654 (11)	−0.0154 (8)	−0.0069 (8)	−0.0198 (9)
C1	0.0621 (14)	0.0655 (14)	0.0918 (19)	−0.0265 (12)	−0.0169 (13)	−0.0299 (14)
C2	0.0538 (13)	0.0547 (13)	0.098 (2)	−0.0278 (11)	−0.0148 (12)	−0.0138 (13)
C3	0.0854 (18)	0.0705 (16)	0.0681 (16)	−0.0395 (14)	−0.0169 (13)	−0.0183 (13)
C4	0.0441 (11)	0.0593 (13)	0.0698 (14)	−0.0252 (10)	−0.0098 (10)	−0.0090 (11)
C5	0.0694 (14)	0.0562 (12)	0.0603 (13)	−0.0317 (11)	−0.0142 (10)	−0.0090 (10)
C6	0.0455 (11)	0.0521 (11)	0.0573 (11)	−0.0198 (9)	−0.0103 (9)	−0.0146 (9)
C7	0.0388 (10)	0.0511 (11)	0.0605 (12)	−0.0163 (9)	−0.0125 (9)	−0.0113 (10)
C8	0.0528 (12)	0.0514 (12)	0.0548 (11)	−0.0165 (10)	−0.0091 (9)	−0.0167 (10)
C9	0.0475 (11)	0.0495 (11)	0.0516 (11)	−0.0183 (9)	−0.0069 (9)	−0.0085 (9)
C10	0.0619 (14)	0.0684 (15)	0.0903 (18)	−0.0237 (12)	−0.0170 (13)	−0.0361 (15)
C11	0.0457 (11)	0.0484 (11)	0.0490 (10)	−0.0169 (9)	−0.0096 (8)	−0.0117 (9)
C12	0.0660 (16)	0.0835 (19)	0.121 (3)	−0.0422 (15)	0.0002 (16)	−0.0455 (19)
C13	0.0544 (14)	0.0774 (17)	0.108 (2)	−0.0333 (13)	0.0044 (15)	−0.0450 (17)
C14	0.0738 (17)	0.0558 (14)	0.0770 (17)	−0.0174 (13)	−0.0090 (15)	−0.0272 (14)

### Geometric parameters (Å, °)

**Table:**

Cl1—C5	1.735 (2)	C6—C11	1.473 (3)
Cl2—C1	1.735 (3)	C6—C13	1.500 (3)
O1—C9	1.262 (2)	C7—C9	1.490 (3)
O2—C8	1.310 (3)	C8—C11	1.383 (3)
O2—H10	0.91 (3)	C8—C10	1.483 (3)
O3—N1	1.413 (2)	C9—C11	1.432 (3)
O3—C14	1.429 (3)	C10—C12	1.509 (4)
N1—C6	1.284 (3)	C10—H8	1.03 (3)
C1—C2	1.366 (4)	C10—H11	1.00 (3)
C1—C3	1.373 (4)	C12—C13	1.497 (4)
C2—C4	1.373 (3)	C12—H7	0.99 (3)
C2—H3	0.90 (3)	C12—H14	1.12 (4)
C3—C5	1.381 (3)	C13—H2	0.91 (3)
C3—H6	0.96 (3)	C13—H15	1.13 (4)
C4—C7	1.384 (3)	C14—H4	0.99 (3)
C4—H1	0.99 (2)	C14—H5	0.94 (3)
C5—C7	1.382 (3)	C14—H9	1.00 (3)
			
C8—O2—H10	104.4 (19)	C11—C9—C7	122.91 (18)
N1—O3—C14	107.48 (18)	C8—C10—C12	110.6 (2)
C6—N1—O3	110.89 (17)	C8—C10—H8	112.0 (19)
C2—C1—C3	121.8 (2)	C12—C10—H8	111.5 (18)
C2—C1—Cl2	119.0 (2)	C8—C10—H11	112.8 (19)
C3—C1—Cl2	119.2 (2)	C12—C10—H11	108.9 (18)
C1—C2—C4	119.2 (2)	H8—C10—H11	101 (2)
C1—C2—H3	122.8 (18)	C8—C11—C9	118.71 (18)
C4—C2—H3	118.0 (18)	C8—C11—C6	118.12 (18)
C1—C3—C5	118.0 (2)	C9—C11—C6	123.15 (18)
C1—C3—H6	122.7 (17)	C13—C12—C10	111.8 (3)
C5—C3—H6	119.3 (17)	C13—C12—H7	107.1 (18)
C2—C4—C7	121.2 (2)	C10—C12—H7	111.0 (17)
C2—C4—H1	120.3 (14)	C13—C12—H14	105 (2)
C7—C4—H1	118.5 (14)	C10—C12—H14	112 (2)
C3—C5—C7	121.9 (2)	H7—C12—H14	109 (3)
C3—C5—Cl1	118.64 (19)	C12—C13—C6	113.5 (2)
C7—C5—Cl1	119.48 (17)	C12—C13—H2	110.8 (19)
N1—C6—C11	116.84 (18)	C6—C13—H2	106.2 (18)
N1—C6—C13	123.33 (19)	C12—C13—H15	102.1 (19)
C11—C6—C13	119.61 (19)	C6—C13—H15	106 (2)
C5—C7—C4	117.9 (2)	H2—C13—H15	118 (3)
C5—C7—C9	123.08 (19)	O3—C14—H4	107.5 (19)
C4—C7—C9	119.0 (2)	O3—C14—H5	105.9 (18)
O2—C8—C11	122.27 (19)	H4—C14—H5	111 (2)
O2—C8—C10	115.41 (19)	O3—C14—H9	108.2 (16)
C11—C8—C10	122.29 (19)	H4—C14—H9	114 (3)
O1—C9—C11	121.04 (19)	H5—C14—H9	110 (2)
O1—C9—C7	116.05 (18)		

### Hydrogen-bond geometry (Å, °)

**Table:**

*D*—H···*A*	*D*—H	H···*A*	*D*···*A*	*D*—H···*A*
O2—H10···O1	0.91 (3)	1.64 (3)	2.485 (2)	152 (3)
C4—H1···O1^i^	0.99 (2)	2.51 (2)	3.347 (3)	143 (2)
C2—H3···O2^ii^	0.91 (4)	2.57 (3)	3.438 (3)	160 (3)

Symmetry codes: (i) −*x*+1, −*y*+2, −*z*; (ii) *x*, *y*+1, *z*.

## References

[bb1] Beddoes, R. L., Kearney, T., Jackson, A. & Joule, J. A. (1992). *Acta Cryst.* C**48**, 1444–1446.

[bb2] Bruker (2003). *SAINT* and *SMART* Bruker AXS Inc., Madison, Wisconsin, USA.

[bb3] Huang, M.-L., Zou, J.-W., Yang, D.-Y., Ning, B.-Z., Shang, Z.-C. & Yu, Q.-S. (2002). *J. Mol. Struct. (THEOCHEM)*, **589–590**, 321–328.

[bb4] Knudsen, C. G. (1988). US Patent 4 775 411.

[bb5] Rubinov, D. B., Rubinova, I. L. & Akhrem, A. A. (1999). *Chem. Rev.* **99**, 1047–1065. 10.1021/cr9600621 11848999

[bb6] Sheldrick, G. M. (2001). *SADABS* University of Göttingen, Germany.

[bb7] Sheldrick, G. M. (2008). *Acta Cryst.* A**64**, 112–122. 10.1107/S0108767307043930 18156677

